# Genotypic Homogeneity of Multidrug Resistant *S*. Typhimurium Infecting Distinct Adult and Childhood Susceptibility Groups in Blantyre, Malawi

**DOI:** 10.1371/journal.pone.0042085

**Published:** 2012-07-27

**Authors:** Chisomo L. Msefula, Robert A. Kingsley, Melita A. Gordon, Elizabeth Molyneux, Malcolm E. Molyneux, Calman A. MacLennan, Gordon Dougan, Robert S. Heyderman

**Affiliations:** 1 Department of Microbiology, College of Medicine, University of Malawi, Blantyre, Malawi; 2 Malawi-Liverpool-Wellcome Trust Clinical Research Programme (MLW), College of Medicine, University of Malawi, Blantyre, Malawi; 3 Department of Paediatrics, College of Medicine, University of Malawi, Blantyre, Malawi; 4 Wellcome Trust Sanger Institute, Wellcome Trust Genome Campus, Hinxton, Cambridge, United Kingdom; 5 University of Liverpool, Liverpool, United Kingdom; 6 Novartis Vaccines Institute for Global Health S.r.l. (NVGH), Siena, Italy; 7 MRC Centre for Immune Regulation, School of Immunity and Infection, College of Medicine and Dental Sciences, University of Birmingham, Edgbaston, Birmingham, United Kingdom; 8 Department of Gastroenterology, Institute of Translational Medicine, University of Liverpool, Liverpool, United Kingdom; 9 Department of Medicine, College of Medicine, University of Malawi, Blantyre, Malawi; Wadsworth Center, New York State Dept. Health, United States of America

## Abstract

Nontyphoidal *Salmonella* (NTS) serovars are a common cause of bacteraemia in young children and HIV-infected adults in Malawi and elsewhere in sub-Saharan Africa. These patient populations provide diverse host-immune environments that have the potential to drive bacterial adaptation and evolution. We therefore investigated the diversity of 27 multidrug resistant (MDR) *Salmonella* Typhimurium strains isolated over 6 years (2002–2008) from HIV-infected adults and children and HIV-uninfected children. Sequence reads from whole-genome sequencing of these isolates using the Illumina GA platform were mapped to the genome of the laboratory strain *S*. Typhimurium SL1344 excluding homoplastic regions that contained prophage and insertion elements. A phylogenetic tree generated from single nucleotide polymorphisms showed that all 27 strains clustered with the prototypical MDR strain D23580. There was no clustering of strains based on host HIV status or age, suggesting that these susceptible populations acquire *S*. Typhimurium from common sources or that isolates are transmitted freely between these populations. However, 7/14 of the most recent isolates (2006/2008) formed a distinct clade that branched off 22 SNPs away from the cluster containing earlier isolates. These data suggest that the MDR bacterial population is not static, but is undergoing microevolution which might result in further epidemiology change.

## Introduction

Nontyphoidal *Salmonella* serovars (NTS) have been a prominent cause of potentially fatal bacteraemia in Malawi and elsewhere in sub-Saharan Africa for more than fifteen years [1], [2], [3], [4]. The majority of isolates in this epidemic are multidrug resistant (MDR) (ampicillin, chloramphenicol and cotrimoxazole), leaving limited therapeutic options for case management [5], [6]. Reports of ceftriaxone and ciprofloxacin resistant *Salmonella* Typhimurium are now emerging from South Africa, emphasising the paramount importance of developing appropriate public health prevention strategies [7]. A vaccine approach to the prevention of NTS bacteraemia will require both an improved understanding of the mechanisms of protective immunity [8], [9], [10], [11] and of the genetic diversity of the circulating invasive strains [12].

In sub-Saharan Africa, invasive NTS particularly affects young children, frequently in association with malnutrition, malaria, severe anaemia and/or HIV infection; and adults almost exclusively with marked HIV-associated immunosuppression [4], [13], [14]. Acquisition of *Salmonella* antibody in children is thought to be protective against bacteraemia [10], while in HIV-infected adults there appears to be dysregulated humoral immunity due to production of antibodies that block bactericidal killing of NTS [9], and dysregulated cellular immunity [15], [16], [17]

Until recently, our understanding of the diversity of *Salmonella* strains causing bacteraemia in children and HIV-infected adults in Malawi was based on serovar and antibiotic resistance differences [5]. We have recently determined that the *S*. Typhimurium strains in Malawi and Kenya belong to sequence type ST313, which is rarely isolated outside sub-Saharan Africa. Whole genome sequencing of epidemic ST313 strains identified a distinct prophage repertoire and a composite element encoding MDR genes located on a virulence-associated plasmid (pSLT-BT, EMBL accession number FN432031) [18]. Evidence of genome degradation, including pseudogene formation and chromosomal deletions found in these strains, suggests that these *S*. Typhimurium strains may have become human adapted [19], [20], [21]. Similar genomic degradation has been identified in host-restricted *Salmonella* serovars such as *S*. Typhi, *S*. Paratyphi A and *S*. Gallinarum [22], [23], [24].

Experimental studies with *E. coli* raise the possibility that the environment within a host can personalise infecting pathogens [25], [26], [27], [28]. A recent study of recurrent invasive ST313 *S*. Typhimurium disease in HIV-infected Malawian adults has, however, shown no evidence of within-individual microevolution across multiple episodes of recurrent disease over time [29]. In view of the distinct at-risk populations for invasive NTS in sub-Saharan Africa and the potential impact on prevention strategies, we have investigated the hypothesis that host differences imprint on the genotype of ST313 MDR *S*. Typhimurium isolates from HIV-infected adults and children and from HIV-uninfected children.

## Results

**Figure 1:**
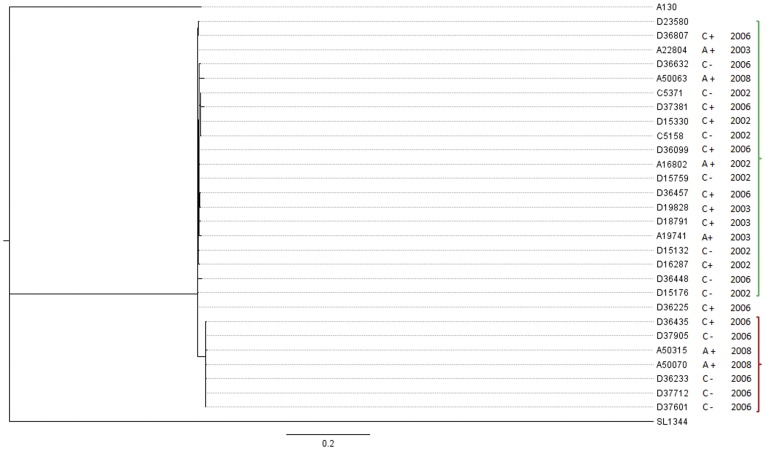
A rectangular phylogram depicting the relatedness of *S*. Typhimurium strains. Phylogeny of invasive multidrug resistant *S*. Typhimurium strains isolated from HIV-infected adults (A+) and children (C+) and HIV-uninfected children (C−) in Blantyre, Malawi, from the period 2002 to 2008 including the epidemic strain D23580. The phylogram also includes the reference gastroenteritis strain SL1344 and the invasive, chloramphenicol-susceptible, pre-epidemic strain A130 as outgroups. A clade consisting isolates from 2006 and 2008 only is highlighted in red. The scale bar value 0.2 represents expected substitutions per site.

**Figure 2:**
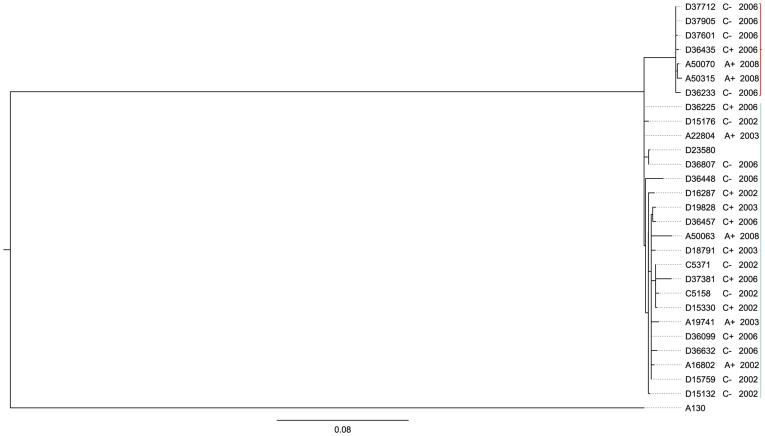
Lack of host specificity and microevolution. A subtree of the phylogram in figure 1 depicting relatedness of invasive multidrug resistant *S*. Typhimurium strains from HIV-infected adults (A+) and children (C+) and HIV-uninfected children (C−) over time (2002–2008) including the epidemic strain D23580. The pre-epidemic chloramphenicol-susceptible *S*. Typhimurium strain A130 is included as an outgroup. The scale bar value 0.08 represents expected substitutions per site.

*Salmonella* Typhimurium isolates that underwent Illumina sequencing comprised representative isolates from HIV-infected adults, HIV-infected children and HIV-uninfected children, from two periods; 2002/2003 and 2006/2008. All 27 isolates from both time periods clustered with the MDR epidemic strain D23580 (Figure 1). Isolates from patients with the same HIV-status or age were no more related to each other than isolates from other susceptibility groups (Figure 2).

There was however distinct clustering within the D23580 clade based on year of isolation. A cluster of seven isolates contained exclusively isolates from 2006/2008. This small cluster was separated by 22 SNPs from the main group of 20 isolates and contained isolates from each of the three susceptibility groups; two from HIV-infected adults, one strain from an HIV-infected child and four from HIV-uninfected children (Figure 2). The topology of the tree indicates that the small cluster containing some of the most recent isolates shares a common ancestor with the larger cluster at the root of the tree, and does not branch off from within the large cluster containing other recent and all the older isolates. The maximum SNP distance within the main group of 20 isolates was fifteen to a 2008 blood isolate A50063. The tree pattern suggests limited accumulation of SNPs over time in the sampled *S*. Typhimurium.

In order to determine whether isolates from distinct susceptibility groups or dates of isolation had distinct genome features we carried out *de novo* assembly of short read sequence and ordered the contiguous sequence using the *S*. Typhimurium D23580 genome sequence and plasmid pSLT-BT from *S.* Typhimurium D23580. All 27 draft genomes were assembled and compared using SNPsFinder [30] and Artemis comparison tool (ACT) [31]. Three SNPs located within intergenic space/noncoding regions, 12 non-synonymous and 2 synonymous SNPs distinguished the 2006/2008 subgroup of strains from the large cluster that includes D23580 (Table 1). Differences between the populations include amino acid substitutions in an arginine exporter protein and in enzymes pyruvate dehydrogenase, fumarate hydratase and nitrate reductase (Table 1). Arginine is essential in the induction of inducible nitric oxide synthase (iNOS) but also promotes the growth of bacterial pathogens [32], [33]. Nitrate reductase contributes to the intracellular survival of *Salmonella*. Pyruvate dehydrogenase and fumarate hydratase are important in the production of energy for the bacterial cell activities [34], [35]. There was no evidence of further genome degradation in the seemingly diverging sub-population except for one non-sense mutation in an open reading frame coding for xylanase/chitin deacetylase (Table 1).

**Table 1:** Single nucleotide polymorphisms unique to strains in the 2006/2008 clade.

STM_MAL	STM	Description	LT2	D23580+19 other strains	2006/2008 strains	Amino acid	gene
4573	179	xylanase/chitin deacetylase	G	G	A	Trp→stop	*yadE*
39	285	inner membrane protein	G	G	A	Ala→Thr	-
624	762	fumarate hydratase	G	G	T	Leu→Ile	-
756	935	pyruvate dehydrogenase	C	T	C	His→Arg	*poxB*
1383	1578	nitrate reductase 2 beta subunit	C	C	T	Arg→Cys	*narY*
1403	1598	regulatory protein	G	A	G	No change	-
2016	2150	outer membrane protein	A	A	T	Asn→Lys	*yehB*
2136	2277	ribonucleotide-diphosphate reductase subunit alpha	T	T	C	Val→Ala	*nrdA*
2170	2312	hypothetical protein	C	T	C	Ile→Val	-
		intergenic space or other non-protein-coding region	G	A	G	-	-
		intergenic space or other non-protein-coding region	T	T	A	-	-
2847	3046	hemolysin	T	T	C	No change	*yqfA*
2865	3066	arginine exporter protein	C	T	C	Glu→Gly	*yggA*
		intergenic space or other non-protein-coding region	A	A	G	-	-
3337	3509	carboxylesterase BioH	A	A	C	Trp→Gly	*bioH*
3719	3899	ATP-dependent protease	G	G	A	Val→Ala	*yifB*
4596	202	glutamate-1-semialdehyde aminotransferase	T	T	C	Asn→Ser	*hemI*

Single nucleotide polymorphisms relative to *S*. Typhimurium strains LT2 and D23580, distinguishing the seven strains of the 2006/2008 lineage from the large cluster of MDR strains (19) and the corresponding amino acid changes. STM and STM_Mal are systematic identification for coding sequences in LT2 and D23580.

Trp – tryptophan, Gln – glutamine, Arg – arginine, Ala – alanine, Thr – threonine, Leu – leucine, Ile – isoleucine, His – histidine, Cys – cysteine, Asn – asparagine, Gly – glycine.

The multidrug resistance locus encoded within a Tn*21*-like element on plasmid pSLT-BT from strains in the 2006/2008 subgroup is similar to the integron earlier characterised in *S*. Typhimurium D23580. Sequence reads of all 27 genomes were mapped to the sequence of the 117 kb plasmid pSLT-BT. Average percent coverage of plasmid pSLT-BT was 95% excluding sequence reads from one strain with a low percent coverage of 12% (Table S1). Assembled plasmid sequences showed that 5% of the genome that was not mapped is probably due to repeat regions within the Tn*21*-like element where sequence gaps exist. Mapping of sequence reads to pSLT-BT generated two SNP loci distributed between two isolates, D36435 from the 2006/2008 subgroup and D36807 from the large cluster of 20 isolates. The two SNPs lie within a putative resolvase coding sequence flanking multidrug resistance loci in both strains. Comparison of assembled plasmid sequences from the 2006/2008 subgroup to pSLT-BT using ACT demonstrated colinearity and synteny (Figure S1).

## Discussion

We addressed the hypothesis that the genotypic composition of populations of multidrug resistant *S*. Typhimurium strains in Malawi will be influenced by factors which are responsible for the host's susceptibility to invasive NTS infection – the principal factors being HIV infection and young age. Phylogenetic analysis revealed a high degree of genetic relatedness of MDR isolates (Figure 2). Clustering of *S*. Typhimurium strains in the phylogenetic tree did not show host preference between HIV-uninfected children and HIV-infected adults. It appears therefore that these strains have not undergone selection or adaptation to the different susceptible host groups. Selection pressures in the affected children and HIV-infected adults may be similar. Adaptive mutations have been identified in experimental models where mutations in *E. coli* isolates passaged through human volunteers could be identified with a particular infected host [25]. This was not the case in our different NTS affected groups. The absence of genotypes associated with a susceptible group does not entirely rule out the possibility of phenotypic adaptation of *S*. Typhimurium strains to the different hosts. Maestroni et al have recently demonstrated regulation of fitness genes in *S.* Typhimurium which enhance virulence after passage through mice without any apparent mutations [36]. It is possible that over a longer period of time such adaptations to different human hosts will emerge.

The high level of genetic relatedness among *S*. Typhimurium isolates from children and HIV-infected adults has implications for strategies to determine the source and mode of transmission and for development of interventions such as a vaccine. MDR *S*. Typhimurium in Malawi account for 90% of all NTS bacteraemia isolates but the source and modes of transmission have not being identified. Our findings suggest that both children and HIV-infected adults are infected by strains emanating from similar sources. Previous studies in Kenya and the Gambia have suggested that human-to-human transmission occurs in invasive NTS disease, since similar isolates could not be found in zoonotic and environmental sources [37], [38]. A majority of the strains in the phylogenetic tree clustered with the fully sequenced and annotated invasive *S*. Typhimurium strain D23580. We have previously shown a reduced genome in D23580 in comparison to gastroenteritis strains, a finding consistent with what we might expect of strains undergoing host adaptation. If *S*. Typhimurium strains in Malawi are circulating between HIV-infected adults and children, it will be important to determine which one of the susceptible groups is the main reservoir.

Multidrug resistant *S*. Typhimurium strains and other NTS serovars are not limited to Malawi; they are spread across sub-Saharan Africa [1], [3], [5], [7]. Understanding the genetic diversity of these strains is key to developing a much needed vaccine as there are limited antibiotic choices against NTS infections. We have found that *S.* Typhimurium strains causing infection in adults and children are genetically similar suggesting that suitable antigenic targets for a vaccine providing protection to both children and HIV-infected adults might be identified. This type of study must be expanded to characterise diversity of other serovar populations in Malawi including *S*. Enteritidis – the second most common NTS bacteraemia isolate – and serovars in other parts of sub-Saharan Africa to ensure adequate coverage by candidate vaccines.

We have determined that 7/27 closely related strains of *S.* Typhimurium from the more recent period between 2006 and 2008 formed a separate clade suggesting a complex epidemiology involving microevolution over time. This divergence has some similarities to the epidemic increase of multidrug resistant *S*. Typhimurium strains between 2001 and 2002 [5] but the 2006/2008 divergent strains are closely related to the predominant type strain. The seemingly diverging subcluster of strains could be a reflection of much wider genetic variation that has not been fully captured in the characterised sample size in this study. The longest SNP distance within the large cluster of strains is 15 SNPs to a tip with a single isolate from 2008. This genetic distance is seven SNPs less than the distance distinguishing the 2006/2008 group of seven strains. Therefore it will be necessary to investigate a larger sample size than the present 27 strains to ascertain further the level of genetic diversity in the population of multidrug resistant *S*. Typhimurium in Malawi. Single nucleotide polymorphisms that distinguish the 2006/2008 sub-population (Table 1) include regions that previously have been shown to impact anaerobic intracellular growth of bacteria. Paiva et al have demonstrated that mutations in nitrate reductase and fumarate reductase genes attenuate the intracellular growth of *Salmonella* Gallinarum in chickens [39]. Further investigations to understand what these single nucleotide polymorphisms mean to the clinical presentation in both HIV-infected adults and children, and to the epidemiology of *S*. Typhimurium strains over time are warranted. It will also be important to determine the selection pressures driving these changes.

In conclusion we have demonstrated the genotypic homogeneity of MDR *S*. Typhimurium strains isolated from HIV-infected adults and children and from HIV-uninfected children, indicating that similar strains are circulating between the distinct susceptibility groups. Homogeneity of the strains suggests that adults and children may have the same sources of infection. We have shown that microevolution of MDR *S*. Typhimurium strains has occurred in Malawi within the past decade. Molecular tools are now available to study the biology of these pathogens and to explore mechanisms that influence clinical presentation and epidemiology.

## Materials and Methods

### Bacterial Isolates

We investigated 27 invasive multidrug resistant *S*. Typhimurium strains isolated from blood or cerebrospinal fluid during the period 2002 to 2008 (Table 2). As described previously, these isolates were obtained as part of routine surveillance for invasive bacterial infection amongst children and adults presenting with a febrile illness to Queen Elizabeth Central Hospital (QECH), Blantyre Malawi [5]. Bacterial strains were selected randomly from 234 *S*. Typhimurium isolated from blood or cerebrospinal fluid of children and adults who participated in three studies in 2002, 2006 and 2008 at QECH and their HIV-sero status was known. Twenty-one strains were from 10/65 HIV-infected and 11/72 HIV-uninfected children. Six strains were selected from a group of 97 isolates from HIV-infected adults. Bacterial culture and antibiotic sensitivity testing were carried out using standard previously described protocols [5].

**Table 2:** List of sequenced invasive MDR *S*. Typhimurium strains (2002–2008).

Isolate	Year	Sero status	source	sex	CN	CRO	C	AMP	SXT	CIP
D15132	2002	HIV−	blood	F	S	S	R	R	R	S
D15176	2002	HIV−	blood	M	S	S	R	R	R	S
D15759	2002	HIV−	blood	F	S	S	R	R	R	S
C5158	2002	HIV−	CSF*	M	S	S	R	R	R	S
C5371	2002	HIV−	CSF*	M	S	S	R	R	R	S
D15330	2002	HIV+	blood	F	S	S	R	R	R	S
D16287	2002	HIV+	blood	F	S	S	R	R	R	S
A16802	2002	HIV+	blood	F	S	S	R	R	R	S
D18791	2003	HIV+	blood	M	S	S	R	R	R	S
D19828	2003	HIV+	blood	M	S	S	R	R	R	S
A19741	2003	HIV+	blood	F	S	S	R	R	R	S
A22804	2003	HIV+	blood	M	S	S	R	R	R	S
D36225	2006	HIV+	blood	F	S	S	R	R	R	S
D36099	2006	HIV+	blood	M	S	S	R	R	R	S
D36435	2006	HIV+	blood	F	S	S	R	R	R	S
D36457	2006	HIV+	blood	M	S	S	R	R	R	S
D36807	2006	HIV+	blood	M	S	S	R	R	R	S
D37381	2006	HIV+	blood	M	S	S	R	R	R	S
D36233	2006	HIV−	blood	F	S	S	R	R	R	S
D36448	2006	HIV−	blood	F	S	S	R	R	R	S
D36632	2006	HIV−	blood	F	S	S	R	R	R	S
D37712	2006	HIV−	blood	F	S	S	R	R	R	S
D37905	2006	HIV−	blood	M	S	S	R	R	R	S
D37601	2006	HIV−	blood	F	S	S	R	R	R	S
A50063	2008	HIV+	blood	M	S	S	R	R	R	S
A50070	2008	HIV+	blood	M	S	S	R	R	R	S
A50315	2008	HIV+	blood	M	S	S	R	R	R	S

Invasive multidrug resistant *S*. Typhimurium strains from Malawian adults and children and corresponding resistance profiles. (CN = gentamicin, CRO = ceftriaxone, C = chloramphenicol, Amp = ampicillin, SXT = cotrimoxazole, CIP = ciprofloxacin).

* CSF = cerebrospinal fluid.

### DNA preparation and Sequence alignment

Genomic DNA was extracted using the Wizard Genome DNA purification kit (Promega, USA) according to the manufacturer's instructions. Genomic DNA sequencing was carried out using the Illumina GA platform (Illumina, UK) in groups of 12 index tagged pools in 54 cycle runs at the Wellcome Trust Sanger Institute, UK. Sequence reads were mapped to plasmid pSLT-BT sequence and to the gastroenteritis *S.* Typhimurium strain SL1344 [40] genome sequence excluding highly variable prophage elements. Prophage elements and repeat sites constituting ∼7% of the reference SL1344 genome were excluded using repeatfinding programmes nucmer, REPeuter and repeat-match [41], [42], [43], [44]. Reads were mapped using the Burrows-Wheeler Aligner software (BWA) with a minimum read depth of 4 [45]. SNPs were identified using mpileup and samtools and filtered with a minimum mapping quality to call a SNP of 30 and a SNP/mapping quality ratio cut-off of 0.75 [29], [46], [47]. A total of 1159 single nucleotide polymorphisms (SNP) sites were identified across the chromosome with an average percent coverage of 94% of the SL1344 sequence (Table S2). The sequence data for all 27 *S*. Typhimurium were submitted to the European Read Archive under submission accession number ERA015722 (http://www.ebi.ac.uk/ena/data/view/ERA015722) and run accession numbers listed in Table S3.

### Phylogeny

A maximum likelihood phylogram was generated based on 1159 single nucleotide polymorphism (SNPs) sites across the chromosome using RAxML v7.0.4 [48] with GTRGAMMA model of evolution and 100 bootstrap replicates. The phylogram was viewed in FigTree (available at http://tree.bio.ed.ac.uk/software/figtree/). The phylogram included the already sequenced and annotated epidemic multidrug resistant strain D23580 EMBL accession number FN424405 and the draft genome of the pre-epidemic and chloramphenicol susceptible *S.* Typhimurium strain A130, accession number ERA000075 [18]. A130 was isolated from blood in an adult patient in 1997 at QECH, Blantyre, Malawi.

### Genome assembly and comparison of plasmid and chromosomal sequences

Genome assembly was conducted using VelvetOptimiser.pl [49] to assemble short read sequences and abacas.pl to order the contigs [50].

A file of SNPs identified by mapping using the Burrows-Wheeler Aligner software was opened in the Artemis file of the reference strain SL1344 under comparison to assembled sequences and the annotated strain D23580, to identify any changes in amino acids. Assembled sequences were compared using the Artemis comparison tool (ACT) [31] and also uploaded on to SNPSFinder together with the fully-sequenced and annotated *S*. Typhimurium strains LT2 (EMBL accession number AE006468) and D23580 [30].

## Supporting Information

Figure S1 **Similarity of Tn** ***21*** **-like sequence from pSLT-BT to assembled sequences.** Artemis comparison tool generated figures A and B depicting sequence homology of the Tn*21*-like sequence (green feature) from virulence plasmid pSLT-BT to assembled plasmid sequences from the seven strains in the 2006/2008 cluster. Antibiotic resistance genes (*cat*- chloramphenicol, *blaT*- betalactamse, *SulI* and *SulI*- sulphonamide, *dhfrI*- trimethoprim, *aadA*- aminoglycoside and *StrA* and *StrB*- streptomycin) and quartenary ammonium compound resistance gene (*qacE*) are present in all six strains. BLASTN matches are shown as red bands. White spaces are gaps in these draft sequences.
(TIF)

Table S1 **Short sequence read mapping to virulence plasmid pSLT-BT sequence.**
(DOCX)

Table S2 **Mapping to** ***S*** **. Typhimurium SL1344 chromosome and identified single nucleotide polymorphisms.**
(DOCX)

Table S3 **Accession numbers (** http://www.ebi.ac.uk/ena/data/view/ERA015722 **).**
(DOCX)

## References

[1] Sow AG, Wane AA, Diallo MH, Boye CS, Aidara-Kane A (2007) Genotypic characterization of antibiotic-resistant Salmonella enteritidis isolates in Dakar, Senegal. J Infect Dev Ctries 1: 284–288. 19734606

[2] Feasey NA, Archer BN, Heyderman RS, Sooka A, Dennis B, et al (2010) Typhoid fever and invasive nontyphoid salmonellosis, Malawi and South Africa. Emerg Infect Dis 16: 1448–1451. 20735930 10.3201/eid1609.100125 PMC3294972

[3] Kariuki S, Revathi G, Kariuki N, Kiiru J, Mwituria J, et al (2006) Invasive multidrug-resistant non-typhoidal Salmonella infections in Africa: zoonotic or anthroponotic transmission? J Med Microbiol 55: 585–591. 16585646 10.1099/jmm.0.46375-0

[4] Graham SM, Walsh AL, Molyneux EM, Phiri AJ, Molyneux ME (2000) Clinical presentation of non-typhoidal Salmonella bacteraemia in Malawian children. Trans R Soc Trop Med Hyg 94: 310–314. 10975008 10.1016/s0035-9203(00)90337-7

[5] Gordon MA, Graham SM, Walsh AL, Wilson L, Phiri A, et al (2008) Epidemics of invasive Salmonella enterica serovar enteritidis and S. enterica Serovar typhimurium infection associated with multidrug resistance among adults and children in Malawi. Clin Infect Dis 46: 963–969. 18444810 10.1086/529146

[6] Kariuki S, Revathi G, Kariuki N, Muyodi J, Mwituria J, et al (2005) Increasing prevalence of multidrug-resistant non-typhoidal salmonellae, Kenya, 1994–2003. Int J Antimicrob Agents 25: 38–43. 15620824 10.1016/j.ijantimicag.2004.08.015

[7] Usha G, Chunderika M, Prashini M, Willem SA, Yusuf ES (2008) Characterization of extended-spectrum beta-lactamases in Salmonella spp. at a tertiary hospital in Durban, South Africa. Diagn Microbiol Infect Dis 62: 86–91. 18513912 10.1016/j.diagmicrobio.2008.04.014

[8] Gondwe EN, Molyneux ME, Goodall M, Graham SM, Mastroeni P, et al (2010) Importance of antibody and complement for oxidative burst and killing of invasive nontyphoidal Salmonella by blood cells in Africans. Proc Natl Acad Sci U S A 107: 3070–3075. 20133627 10.1073/pnas.0910497107 PMC2840319

[9] MacLennan CA, Gilchrist JJ, Gordon MA, Cunningham AF, Cobbold M, et al (2010) Dysregulated humoral immunity to nontyphoidal Salmonella in HIV-infected African adults. Science 328: 508–512. 20413503 10.1126/science.1180346 PMC3772309

[10] MacLennan CA, Gondwe EN, Msefula CL, Kingsley RA, Thomson NR, et al (2008) The neglected role of antibody in protection against bacteremia caused by nontyphoidal strains of Salmonella in African children. J Clin Invest 118: 1553–1562. 18357343 10.1172/JCI33998 PMC2268878

[11] Nyirenda TS, Seeley AE, Mandala WL, Drayson MT, MacLennan CA (2010) Early interferon-gamma production in human lymphocyte subsets in response to nontyphoidal Salmonella demonstrates inherent capacity in innate cells. PLoS One 5: e13667. 21048923 10.1371/journal.pone.0013667 PMC2965112

[12] Clemens JD (2009) Meeting on establishment of consortium to study invasive Salmonelloses in Sub-Saharan Africa. Emerg Infect Dis 15: e2. 19624911 10.3201/eid1507.090416 PMC5836488

[13] Brent AJ, Oundo JO, Mwangi I, Ochola L, Lowe B, et al (2006) Salmonella bacteremia in Kenyan children. Pediatr Infect Dis J 25: 230–236. 16511385 10.1097/01.inf.0000202066.02212.ff

[14] Gordon MA, Banda HT, Gondwe M, Gordon SB, Boeree MJ, et al (2002) Non-typhoidal salmonella bacteraemia among HIV-infected Malawian adults: high mortality and frequent recrudescence. AIDS 16: 1633–1641. 12172085 10.1097/00002030-200208160-00009

[15] Gordon MA, Gordon SB, Musaya L, Zijlstra EE, Molyneux ME, et al (2007) Primary macrophages from HIV-infected adults show dysregulated cytokine responses to Salmonella, but normal internalization and killing. AIDS 21: 2399–2408. 18025876 10.1097/QAD.0b013e3282f25107

[16] Gordon MA, Kankwatira AM, Mwafulirwa G, Walsh AL, Hopkins MJ, et al (2010) Invasive non-typhoid salmonellae establish systemic intracellular infection in HIV-infected adults: an emerging disease pathogenesis. Clin Infect Dis 50: 953–962. 20180702 10.1086/651080

[17] Schreiber F, Lynn DJ, Houston A, Peters J, Mwafulirwa G, et al (2011) The human transcriptome during nontyphoid Salmonella and HIV coinfection reveals attenuated NFkappaB-mediated inflammation and persistent cell cycle disruption. J Infect Dis 204: 1237–1245. 21917897 10.1093/infdis/jir512 PMC3173506

[18] Kingsley RA, Msefula CL, Thomson NR, Kariuki S, Holt KE, et al (2009) Epidemic multiple drug resistant Salmonella Typhimurium causing invasive disease in sub-Saharan Africa have a distinct genotype. Genome Res 19: 2279–2287. 19901036 10.1101/gr.091017.109 PMC2792184

[19] Sakharkar KR, Dhar PK, Chow VT (2004) Genome reduction in prokaryotic obligatory intracellular parasites of humans: a comparative analysis. Int J Syst Evol Microbiol 54: 1937–1941. 15545414 10.1099/ijs.0.63090-0

[20] Moran NA, Plague GR (2004) Genomic changes following host restriction in bacteria. Curr Opin Genet Dev 14: 627–633. 15531157 10.1016/j.gde.2004.09.003

[21] Chain PS, Carniel E, Larimer FW, Lamerdin J, Stoutland PO, et al (2004) Insights into the evolution of Yersinia pestis through whole-genome comparison with Yersinia pseudotuberculosis. Proc Natl Acad Sci U S A 101: 13826–13831. 15358858 10.1073/pnas.0404012101 PMC518763

[22] Holt KE, Thomson NR, Wain J, Langridge GC, Hasan R, et al (2009) Pseudogene accumulation in the evolutionary histories of Salmonella enterica serovars Paratyphi A and Typhi. BMC Genomics 10: 36. 19159446 10.1186/1471-2164-10-36 PMC2658671

[23] Thomson NR, Clayton DJ, Windhorst D, Vernikos G, Davidson S, et al (2008) Comparative genome analysis of Salmonella Enteritidis PT4 and Salmonella Gallinarum 287/91 provides insights into evolutionary and host adaptation pathways. Genome Res 18: 1624–1637. 18583645 10.1101/gr.077404.108 PMC2556274

[24] Parkhill J, Dougan G, James KD, Thomson NR, Pickard D, et al (2001) Complete genome sequence of a multiple drug resistant Salmonella enterica serovar Typhi CT18. Nature 413: 848–852. 11677608 10.1038/35101607

[25] Zdziarski J, Brzuszkiewicz E, Wullt B, Liesegang H, Biran D, et al (2010) Host imprints on bacterial genomes–rapid, divergent evolution in individual patients. PLoS Pathog 6. 10.1371/journal.ppat.1001078 PMC2928814 20865122

[26] Gordon DM, Stern SE, Collignon PJ (2005) Influence of the age and sex of human hosts on the distribution of Escherichia coli ECOR groups and virulence traits. Microbiology 151: 15–23. 15632421 10.1099/mic.0.27425-0

[27] Sokurenko EV, Chesnokova V, Dykhuizen DE, Ofek I, Wu XR, et al (1998) Pathogenic adaptation of Escherichia coli by natural variation of the FimH adhesin. Proc Natl Acad Sci U S A 95: 8922–8926. 9671780 10.1073/pnas.95.15.8922 PMC21178

[28] Alizon S, Luciani F, Regoes RR (2011) Epidemiological and clinical consequences of within-host evolution. Trends Microbiol 19: 24–32. 21055948 10.1016/j.tim.2010.09.005

[29] Okoro CK, Kingsley RA, Quail MA, Kankwatira AM, Feasey NA, et al (2012) High-resolution single nucleotide polymorphism analysis distinguishes recrudescence and reinfection in recurrent invasive nontyphoidal Salmonella typhimurium disease. Clin Infect Dis 54: 955–963. 22318974 10.1093/cid/cir1032 PMC3297646

[30] Song J, Xu Y, White S, Miller KW, Wolinsky M (2005) SNPsFinder–a web-based application for genome-wide discovery of single nucleotide polymorphisms in microbial genomes. Bioinformatics 21: 2083–2084. 15691853 10.1093/bioinformatics/bti176

[31] Carver TJ, Rutherford KM, Berriman M, Rajandream MA, Barrell BG, et al (2005) ACT: the Artemis Comparison Tool. Bioinformatics 21: 3422–3423. 15976072 10.1093/bioinformatics/bti553

[32] Nicholson B, Manner CK, Kleeman J, MacLeod CL (2001) Sustained nitric oxide production in macrophages requires the arginine transporter CAT2. J Biol Chem 276: 15881–15885. 11278602 10.1074/jbc.M010030200

[33] Das P, Lahiri A, Chakravortty D (2010) Modulation of the arginase pathway in the context of microbial pathogenesis: a metabolic enzyme moonlighting as an immune modulator. PLoS Pathog 6: e1000899. 20585552 10.1371/journal.ppat.1000899 PMC2887468

[34] Sohaskey CD (2008) Nitrate enhances the survival of Mycobacterium tuberculosis during inhibition of respiration. J Bacteriol 190: 2981–2986. 18296525 10.1128/JB.01857-07 PMC2293237

[35] Pei Y, Parreira V, Nicholson VM, Prescott JF (2007) Mutation and virulence assessment of chromosomal genes of Rhodococcus equi 103. Can J Vet Res 71: 1–7. 17193875 PMC1636002

[36] Mastroeni P, Morgan FJ, McKinley TJ, Shawcroft E, Clare S, et al (2011) Enhanced virulence of Salmonella enterica serovar typhimurium after passage through mice. Infect Immun 79: 636–643. 21098099 10.1128/IAI.00954-10 PMC3028859

[37] Dione MM, Ikumapayi UN, Saha D, Mohammed NI, Geerts S, et al (2011) Clonal differences between Non-Typhoidal Salmonella (NTS) recovered from children and animals living in close contact in the Gambia. PLoS Negl Trop Dis 5: e1148. 21655353 10.1371/journal.pntd.0001148 PMC3104961

[38] Kariuki S, Revathi G, Gakuya F, Yamo V, Muyodi J, et al (2002) Lack of clonal relationship between non-typhi Salmonella strain types from humans and those isolated from animals living in close contact. FEMS Immunol Med Microbiol 33: 165–171. 12110478 10.1111/j.1574-695X.2002.tb00587.x

[39] Paiva JB, Filho PR, Pereira EA, et al (2009) The contribution of genes required for anaerobic respiration to the virulence of Salmonella enterica serovar Gallinarum for Chickens. Brazilian Journal of Microbiology 40: 7. 10.1590/S1517-838220090004000035 PMC3768590 24031452

[40] Hoiseth SK, Stocker BA (1981) Aromatic-dependent Salmonella typhimurium are non-virulent and effective as live vaccines. Nature 291: 238–239. 7015147 10.1038/291238a0

[41] Holt KE, Parkhill J, Mazzoni CJ, Roumagnac P, Weill FX, et al (2008) High-throughput sequencing provides insights into genome variation and evolution in Salmonella Typhi. Nat Genet 40: 987–993. 18660809 10.1038/ng.195 PMC2652037

[42] He M, Sebaihia M, Lawley TD, Stabler RA, Dawson LF, et al (2010) Evolutionary dynamics of Clostridium difficile over short and long time scales. Proc Natl Acad Sci U S A 107: 7527–7532. 20368420 10.1073/pnas.0914322107 PMC2867753

[43] Kurtz S, Choudhuri JV, Ohlebusch E, Schleiermacher C, Stoye J, et al (2001) REPuter: the manifold applications of repeat analysis on a genomic scale. Nucleic Acids Res 29: 4633–4642. 11713313 10.1093/nar/29.22.4633 PMC92531

[44] Kurtz S, Phillippy A, Delcher AL, Smoot M, Shumway M, et al (2004) Versatile and open software for comparing large genomes. Genome Biol 5: R12. 14759262 10.1186/gb-2004-5-2-r12 PMC395750

[45] Li H, Durbin R (2009) Fast and accurate short read alignment with Burrows-Wheeler transform. Bioinformatics 25: 1754–1760. 19451168 10.1093/bioinformatics/btp324 PMC2705234

[46] Li H, Handsaker B, Wysoker A, Fennell T, Ruan J, et al (2009) The Sequence Alignment/Map format and SAMtools. Bioinformatics 25: 2078–2079. 19505943 10.1093/bioinformatics/btp352 PMC2723002

[47] Harris SR, Feil EJ, Holden MT, Quail MA, Nickerson EK, et al (2010) Evolution of MRSA during hospital transmission and intercontinental spread. Science 327: 469–474. 20093474 10.1126/science.1182395 PMC2821690

[48] Stamatakis A (2006) RAxML-VI-HPC: maximum likelihood-based phylogenetic analyses with thousands of taxa and mixed models. Bioinformatics 22: 2688–2690. 16928733 10.1093/bioinformatics/btl446

[49] Zerbino DR, Birney E (2008) Velvet: algorithms for de novo short read assembly using de Bruijn graphs. Genome Res 18: 821–829. 18349386 10.1101/gr.074492.107 PMC2336801

[50] Assefa S, Keane TM, Otto TD, Newbold C, Berriman M (2009) ABACAS: algorithm-based automatic contiguation of assembled sequences. Bioinformatics 25: 1968–1969. 19497936 10.1093/bioinformatics/btp347 PMC2712343

