# Supplementary figures and images for: Genotypic Homogeneity of Multidrug Resistant S. Typhimurium Infecting Distinct Adult and Childhood Susceptibility Groups in Blantyre, Malawi
Source: PLoS One. 2012 Jul 27;7(7):e42085. doi: 10.1371/journal.pone.0042085 (PMC3407126; doi:10.1371/journal.pone.0042085)

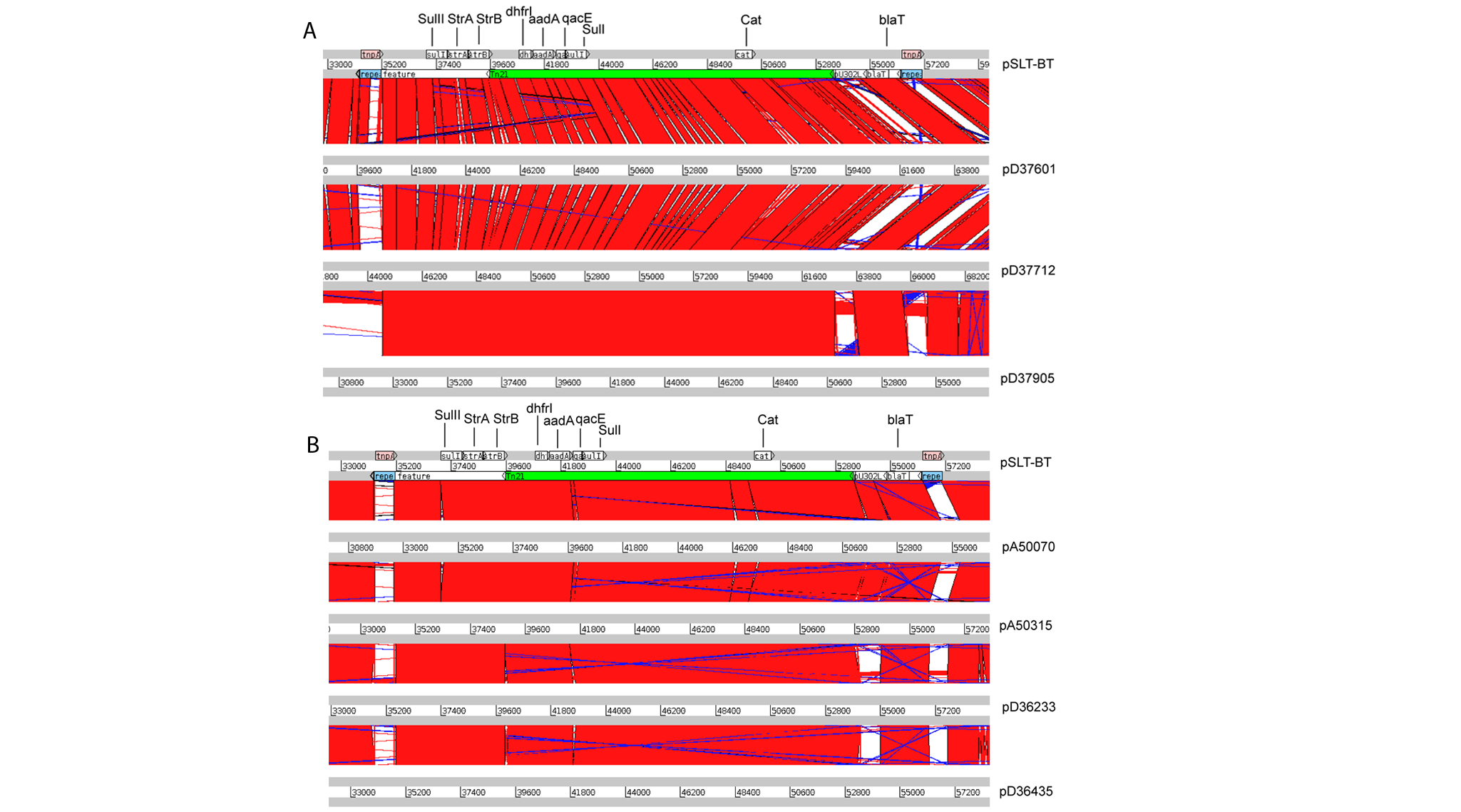

Supplement: Figure S1 — Similarity of Tn 21 -like sequence from pSLT-BT to assembled sequences. Artemis comparison tool generated figures A and B depicting sequence homology of the Tn21-like sequence (green feature) from virulence plasmid pSLT-BT to assembled plasmid sequences from the seven strains in the 2006/2008 cluster. Antibiotic resistance genes (cat- chloramphenicol, blaT- betalactamse, SulI and SulI- sulphonamide, dhfrI- trimethoprim, aadA- aminoglycoside and StrA and StrB- streptomycin) and quartenary ammonium compound resistance gene (qacE) are present in all six strains. BLASTN matches are shown as red bands. White spaces are gaps in these draft sequences. (TIF) [file pone.0042085.s001.tif]
