# Supplementary material for: Genotypic Homogeneity of Multidrug Resistant S. Typhimurium Infecting Distinct Adult and Childhood Susceptibility Groups in Blantyre, Malawi
Source: PLoS One. 2012 Jul 27;7(7):e42085. doi: 10.1371/journal.pone.0042085 (PMC3407126; doi:10.1371/journal.pone.0042085)
Supplement: Table S2 — Mapping to S . Typhimurium SL1344 chromosome and identified single nucleotide polymorphisms. (DOCX) [file pone.0042085.s003.docx]

**Table S2.** Mapping to *S*. Typhimurium SL1344 chromosome and identified single nucleotide polymorphisms.

| **Strain** | **% mapped** | **Mean read depth** | **A > C** | **A > G** | **A > T** | **C > A** | **C > G** | **C > T** | **G > A** | **G > C** | **G > T** | **T > A** | **T > C** | **T > G** | **Total SNPs** |
| --- | --- | --- | --- | --- | --- | --- | --- | --- | --- | --- | --- | --- | --- | --- | --- |
| D15132 | 91.38 | 15 | 31 | 125 | 17 | 46 | 12 | 131 | 139 | 8 | 39 | 15 | 138 | 70 | 731 |
| D18791 | 66.17 | 9 | 28 | 97 | 11 | 37 | 11 | 118 | 106 | 7 | 35 | 13 | 122 | 23 | 608 |
| D19828 | 98.25 | 54 | 35 | 131 | 15 | 48 | 13 | 145 | 146 | 10 | 42 | 15 | 147 | 30 | 777 |
| D36225 | 98.24 | 63 | 34 | 133 | 16 | 50 | 14 | 138 | 144 | 10 | 40 | 15 | 147 | 33 | 774 |
| D15176 | 98.20 | 37 | 34 | 131 | 16 | 49 | 14 | 143 | 148 | 9 | 39 | 15 | 142 | 31 | 771 |
| D15759 | 98.21 | 39 | 35 | 132 | 16 | 48 | 14 | 141 | 147 | 9 | 43 | 15 | 146 | 30 | 776 |
| C5158 | 94.40 | 15 | 32 | 125 | 15 | 47 | 14 | 136 | 141 | 8 | 43 | 15 | 137 | 32 | 745 |
| C5371 | 84.16 | 12 | 31 | 117 | 11 | 45 | 14 | 126 | 126 | 9 | 35 | 14 | 128 | 25 | 681 |
| D15330 | 97.19 | 23 | 34 | 128 | 16 | 47 | 14 | 141 | 146 | 9 | 41 | 14 | 145 | 32 | 767 |
| D16287 | 96.76 | 21 | 33 | 130 | 15 | 49 | 14 | 141 | 144 | 10 | 41 | 15 | 142 | 30 | 764 |
| D36099 | 94.62 | 17 | 34 | 126 | 15 | 47 | 14 | 136 | 145 | 7 | 41 | 14 | 143 | 30 | 752 |
| D37712 | 98.20 | 54 | 34 | 132 | 17 | 50 | 13 | 140 | 147 | 11 | 41 | 16 | 149 | 32 | 782 |
| D37905 | 98.25 | 71 | 36 | 130 | 16 | 49 | 13 | 139 | 146 | 11 | 40 | 16 | 148 | 30 | 774 |
| D37601 | 96.42 | 25 | 33 | 131 | 16 | 48 | 13 | 132 | 142 | 10 | 39 | 16 | 143 | 29 | 752 |
| D36435 | 98.19 | 46 | 35 | 130 | 16 | 50 | 13 | 138 | 146 | 9 | 40 | 16 | 146 | 30 | 769 |
| D36457 | 98.23 | 56 | 34 | 130 | 15 | 48 | 13 | 144 | 146 | 9 | 42 | 15 | 146 | 30 | 772 |
| D36807 | 98.05 | 32 | 34 | 130 | 17 | 48 | 14 | 142 | 149 | 9 | 40 | 14 | 146 | 33 | 776 |
| D37381 | 86.76 | 15 | 30 | 110 | 13 | 48 | 13 | 118 | 123 | 9 | 38 | 14 | 123 | 29 | 668 |
| D36233 | 97.70 | 28 | 34 | 133 | 17 | 49 | 13 | 140 | 147 | 10 | 41 | 16 | 149 | 31 | 780 |
| D36448 | 97.96 | 36 | 34 | 132 | 16 | 50 | 14 | 145 | 149 | 10 | 42 | 15 | 147 | 31 | 785 |
| D36632 | 98.24 | 66 | 34 | 132 | 15 | 48 | 14 | 144 | 148 | 9 | 43 | 15 | 146 | 34 | 782 |
| A16807 | 98.13 | 37 | 34 | 130 | 15 | 49 | 14 | 141 | 148 | 9 | 43 | 14 | 145 | 31 | 773 |
| A19741 | 98.22 | 31 | 35 | 133 | 16 | 49 | 14 | 144 | 149 | 9 | 43 | 15 | 143 | 33 | 783 |
| A22804 | 64.63 | 9 | 24 | 93 | 11 | 42 | 12 | 108 | 107 | 7 | 28 | 11 | 104 | 21 | 568 |
| A50063 | 98.12 | 32 | 36 | 129 | 16 | 48 | 14 | 148 | 150 | 10 | 43 | 16 | 146 | 32 | 788 |
| A50070 | 98.00 | 33 | 34 | 132 | 16 | 49 | 13 | 138 | 145 | 10 | 39 | 15 | 147 | 29 | 767 |
| A50315 | 98.19 | 34 | 35 | 133 | 17 | 49 | 13 | 141 | 146 | 9 | 40 | 16 | 151 | 32 | 782 |
| A130 | 90.17 | 11 | 31 | 118 | 11 | 43 | 11 | 134 | 144 | 7 | 38 | 10 | 141 | 31 | 719 |
| D23580 | 98.06 | 11 | 33 | 126 | 15 | 48 | 14 | 135 | 144 | 9 | 38 | 14 | 144 | 30 | 750 |

Average percent coverage = 94%, reads were mapped with a minimum depth of 4
