# Supplementary material for: Genotypic Homogeneity of Multidrug Resistant S. Typhimurium Infecting Distinct Adult and Childhood Susceptibility Groups in Blantyre, Malawi
Source: PLoS One. 2012 Jul 27;7(7):e42085. doi: 10.1371/journal.pone.0042085 (PMC3407126; doi:10.1371/journal.pone.0042085)
Supplement: Table S3 — Accession numbers ( http://www.ebi.ac.uk/ena/data/view/ERA015722 ). (DOCX) [file pone.0042085.s004.docx]

**Table S3**. Accession numbers (<http://www.ebi.ac.uk/ena/data/view/ERA015722>).

| 4821_1#1 | D15132 | ERR023620 |
| --- | --- | --- |
| 4821_1#10 | D18791 | ERR023621 |
| 4821_1#11 | D19828 | ERR023622 |
| 4821_1#2 | D15176 | ERR023624 |
| 4821_1#3 | D15759 | ERR023625 |
| 4821_1#4 | C5158 | ERR023626 |
| 4821_1#5 | C5371 | ERR023627 |
| 4821_1#8 | D15330 | ERR023630 |
| 4821_1#9 | D16287 | ERR023631 |
| 4821_2#1 | D36099 | ERR023633 |
| 4821_2#10 | D37712 | ERR023634 |
| 4821_2#11 | D37905 | ERR023635 |
| 4821_2#12 | D37601 | ERR023636 |
| 4821_2#2 | D36225 | ERR023637 |
| 4821_2#3 | D36435 | ERR023638 |
| 4821_2#4 | D36457 | ERR023639 |
| 4821_2#5 | D36807 | ERR023640 |
| 4821_2#6 | D37381 | ERR023641 |
| 4821_2#7 | D36233 | ERR023642 |
| 4821_2#8 | D36448 | ERR023643 |
| 4821_2#9 | D36632 | ERR023644 |
| 4821_3#2 | A16802 | ERR023650 |
| 4821_3#5 | A19741 | ERR023653 |
| 4821_3#6 | A22804 | ERR023654 |
| 4821_3#7 | A50063 | ERR023655 |
| 4821_3#8 | A50070 | ERR023656 |
| 4821_3#9 | A50315 | ERR023657 |
